# Photodynamic therapy combined with anti-vascular endothelial growth factor therapy for pachychoroid neovasculopathy

**DOI:** 10.1371/journal.pone.0248760

**Published:** 2021-03-23

**Authors:** Akiko Miki, Sentaro Kusuhara, Tsuyoshi Otsuji, Yu Kawashima, Katsuaki Miki, Hisanori Imai, Makoto Nakamura, Akitaka Tsujikawa

**Affiliations:** 1 Division of Ophthalmology, Department of Surgery, Kobe University Graduate School of Medicine, Kobe, Japan; 2 Department of Ophthalmology, Kansai Medical University Medical Center, Osaka, Japan; 3 Department of Ophthalmology and Visual Sciences, Kyoto University Graduate School of Medicine, Kyoto, Japan; University of Utah (Salt Lake City), UNITED STATES

## Abstract

This multicenter retrospective study was conducted to evaluate the 1-year treatment outcome of photodynamic therapy (PDT) combined with anti-vascular endothelial growth factor (VEGF) therapy for pachychoroid neovasculopathy (PNV). A total of 42 eyes of 42 patients with treatment-naïve PNV who were treated with PDT combined with intravitreal injections of an anti-VEGF agent (ranibizumab or aflibercept) for 1 year. All eyes showed exudative and/or hemorrhagic changes that affected the fovea at baseline. After the initial combination therapy, subfoveal choroidal thickness (SCT) and central retinal thickness (CRT) were significantly reduced and were maintained as such for 12 months (P < 0.01 in SCT and CRT). The best-corrected visual acuity (BCVA) (0.19 ± 0.30 at baseline) significantly improved at 3 months (0.15 ± 0.29, P < 0.05) and further improved at 12 months (0.10 ± 0.30, P < 0.01) when compared to that at baseline. After the initial combination therapy, 32 eyes (76.2%) required no additional treatments for 12 months. The mean number of additional PDT and intravitreal injections of anti-VEGF agents was 0.1 ± 0.3 and 0.9 ± 1.9, respectively. Of the 42 eyes included in this study, 22 eyes (52.4%) had polypoidal lesions at baseline. No significant differences in SCT, CRT, or BCVA were observed at any time points between eyes with and without polypoidal lesions. Of 20 eyes without polypoidal lesions, only 1 eye (5.0%) needed additional treatments. PNV, especially without polypoidal lesions, can be treated effectively with PDT combined with anti-VEGF therapy with few sessions.

## Introduction

Pachychoroid spectrum diseases proposed by Freund [1] are now well accepted as a new concept of macular diseases, which are characterized by a thick choroid and pathologically dilated outer choroidal vessels (pachyvessels) as well as regional choroidal vascular hyperpermeability. They included central serous chorioretinopathy (CSC), pachychoroid pigment epitheliopathy, pachychoroid neovasculopathy (PNV), and polypoidal choroidal vasculopathy (PCV). The pathogenesis of CSC is considered to be a leakage from the pachychoroid through the retinal pigment epithelium (RPE). In PNV, pachychoroid-driven mechanisms induce choroidal neovascularization (CNV), which is characterized as type 1 CNV and polypoidal lesions [2, 3].

Now, anti-vascular endothelial growth factor (VEGF) therapy is a gold standard for neovascular age-related macular degeneration (AMD). However, the efficacy of anti-VEGF therapy for PNV remains controversial [4, 5]. On the other hand, in the clinical setting, photodynamic therapy (PDT) is rarely performed for the treatment of neovascular AMD. However, PDT is one of the standard treatments for CSC. In CSC, a single session of PDT often achieves not only the regression of the serous retinal detachment but also the reduction of the choroidal thickness. In addition, Hata et al. [6] reported that PDT is an effective treatment for PCV, especially with choroidal vascular hyperpermeability (CVH). Recently, the EVEREST II study [7] demonstrated that PDT combined with intravitreal ranibizumab was superior to ranibizumab monotherapy for eyes with PCV in terms of the number of injections and visual outcomes.

Based on these findings, PDT combined with anti-VEGF therapy may be an option for the treatment of PNV. However, information on the efficacy of combination therapy for PNV is limited [8, 9]. In this study, we retrospectively investigated the 1-year treatment outcome of PDT combined with intravitreal injections of anti-VEGF agents for the treatment of PNV that was performed in three university hospitals in Japan.

## Materials and methods

This study was approved by the institutional review boards of Kobe University Hospital, Kyoto University, and Kansai Medical University Medical Center and was conducted in accordance with the tenets of the Declaration of Helsinki.

We retrospectively reviewed the medical records of 42 eyes of 42 patients with treatment-naïve PNV who were treated between January 2012 and March 2019 at Kobe University Hospital, Kyoto University Hospital, or Kansai Medical University Medical Center. Informed consent was obtained by the opt-out method on the center’s website. All eyes showed exudative and/or hemorrhagic changes that affect the fovea, with a visual impairment. The criteria for a diagnosis of PNV were as follows; (1) CNV in the macular area; (2) no drusen or only non-extensive (total area, ≤125 μm circle) hard drusen (≤63 μm) (Age-Related Eye Disease Study level 1,[O] no AMD); (3) clinical and anatomic features of the pachychoroid phenotype, such as reduced fundus tessellation on color fundus photographs, pathologically dilated outer choroidal vessels (pachyvessels) on optical coherence tomography (OCT) and indocyanine green angiography (ICGA) images, and regional CVH on ICGA images. We included also eyes that initially developed CSC with characteristic serous retinal detachments followed by CNV; in this study, four eyes met this criterion. We diagnosed the presence of polypoidal lesions in accordance with a previous report [10]. The exclusion criteria were as follows: (1) severely myopic or hyperopic eyes with refractive errors (spherical equivalent) of more than six diopters; (2) presence of any other ocular diseases that could affect visual acuity, including tilted disc syndrome, dome-shaped macula, and uveitis; (3) history of laser photocoagulation, transpupillary thermotherapy, or intravitreal injections of anti-VEGF agents; and (4) presence of media opacities.

At baseline, each patient received a complete ophthalmologic examination, including slit-lamp examination, dilated fundus examination, and best-corrected visual acuity (BCVA) measurement in a Landolt C chart. Digital fluorescein and indocyanine green angiographies were obtained using the SPECTRALIS OCT system (Heidelberg Spectralis OCT; Heidelberg Engineering GmbH, Heidelberg, Germany). Macular scans were also obtained using the SPECTRALIS OCT system (Heidelberg Spectralis OCT) or RTVue-100 (Optovue, Fremont, CA, USA). Clinical data, including OCT parameters and BCVA at baseline and 1, 3, 6, and 12 months after initial treatment, were recorded.

Central retinal thickness (CRT) was manually measured from the inner neurosensory retina surface to the inner surface of RPE at the foveal center. Subfoveal choroidal thickness (SCT) was also manually measured from the outer surface of RPE to the inner surface of the sclera at the foveal center.

After comprehensive examinations, each patient received an intravitreal injection of ranibizumab (Lucentis; Novartis, Basel, Switzerland) or aflibercept (Eylea; Bayer HealthCare, Berlin, Germany) and standard full PDT with an infusion of verteporfin (Visudyne; Novartis, Basel, Switzerland). After the initial treatment, each patient received dilated fundus examination, BCVA measurement, and OCT examination at each visit. Fluorescein angiography and ICGA were performed, if deemed necessary. Additional treatment was given in eyes with persistent or recurrent exudative change and/or new hemorrhage in the macular area. The choice of additional treatment (an intravitreal anti-VEGF injection or PDT combined with an intravitreal anti-VEGF injection) was determined at the physicians’ discretion. No eyes received any other forms of treatments, such as direct laser photocoagulation, surgical intervention, or steroid.

The decimal BCVA was converted into a logarithm of the minimum angle of resolution (logMAR) for statistical analyses. Differences in BCVA and OCT measurements were analyzed using the Wilcoxon signed-rank test. Mann–Whitney U test was used to compare age, the number of additional treatments, and spot size at baseline and the values of BCVA, CRT, and SCT at each time point between the two groups based on the presence or absence of polypoidal lesions. Statistical comparisons between the two groups were conducted using the chi-square test or Fisher’s exact test for categorical variables. A P-value <0.05 was considered to be statistically significant. Statistical analyses were conducted using the SPSS software, version 25.0 (IBM, Armonk, NY, USA).

## Results

In the current study, 42 eyes (42 patients) with PNV were treated with PDT combined with an intravitreal injection of anti-VEGF agent. Table 1 shows clinical characteristics of the patients. All eyes showed exudative and/or hemorrhagic changes that affected the fovea at baseline.

**Table 1:** Clinical characteristics of patients at baseline.

	All (n = 42)	Without polypoidal lesions (n = 20)	With polypoidal lesions (n = 22)	*P*-value
Age (years)	68.5 ± 11.5	65.4 ± 13.7	71.2 ± 8.6	0.14
Sex (Male/Female)	31/11	12/8	19/3	0.08
logMAR BCVA	0.19 ± 0.30	0.15 ± 0.25	0.23 ± 0.33	0.45
SCT (μm)	316.2 ± 98.7	326.9 ± 106.4	301.9 ± 88.9	0.46
CRT (μm)	286.6 ± 102.0	268.0 ± 44.0	303.5 ± 102.0	0.63

BCVA, best-corrected visual acuity; log MAR, logarithmic minimum angle of resolution; SCT, subfoveal choroidal thickness; CRT, central retinal thickness.

Wilcoxon signed-rank test was used for statistical analysis between eyes with and without polypoidal lesions.

P-value of <0.05 was set as statistically significant.

**Fig 1:**
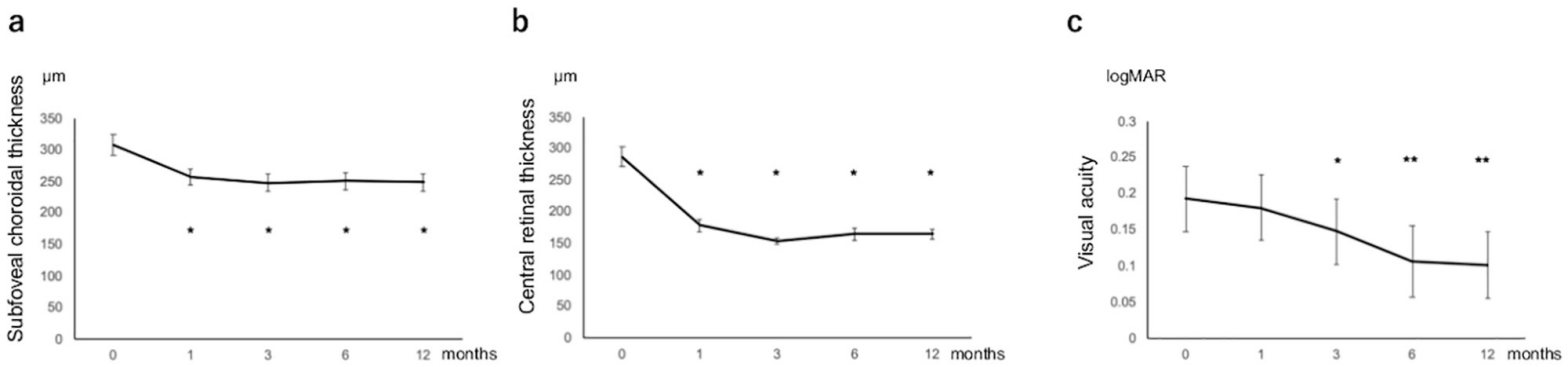
Changes in subfoveal choroidal thickness (a), central retinal thickness (b), and best-corrected visual acuity (c) during the 12-month follow-up period in eyes with pachychoroid neovasculopathy. Each eye was treated with photodynamic therapy combined with intravitreal injections of an anti-vascular endothelial growth factor agent. *P < 0.05, **P < 0.01, compared with baseline values. Data are expressed as mean ± standard error.

Fig 1 shows the changes in SCT, CRT, and BCVA for 12 months. After the initial combination therapy, SCT and CRT achieved a significant reduction and the reduction maintained for 12 months (P < 0.01 in SCT and CRT). In accordance with the reduction of SCT and CRT, BCVA (0.19 ± 0.30 at baseline) significantly improved at 3 months (0.15 ± 0.29, P < 0.05) and further improved at 12 months (0.10 ± 0.30, P < 0.01), compared with that at baseline.

After the initial combination therapy, 32 eyes (76.2%) required no additional treatments for 12 months and 10 eyes (23.8%) received additional treatments. Table 2 shows the number of additional treatments; the mean number of additional PDT and intravitreal injections of anti-VEGF agents was 0.1 ± 0.3 and 0.9 ± 1.9, respectively. None of the patients showed any severe ocular complications, such as massive subretinal hemorrhage, tear of the RPE, or severe vision loss.

**Table 2:** Total number of additional treatments during the 12-month follow-up.

	All (n = 42)	Without polypoidal lesions (n = 20)	With polypoidal lesions (n = 22)	*P*-value
Anti-VEGF	0.9 ± 1.9	0.1 ± 0.4	1.5 ± 2.4	<0.01
PDT	0.1 ± 0.3	0	0.2 ± 0.4	0.05

VEGF, vascular endothelial growth factor; PDT, photodynamic therapy.

**Fig 2:**
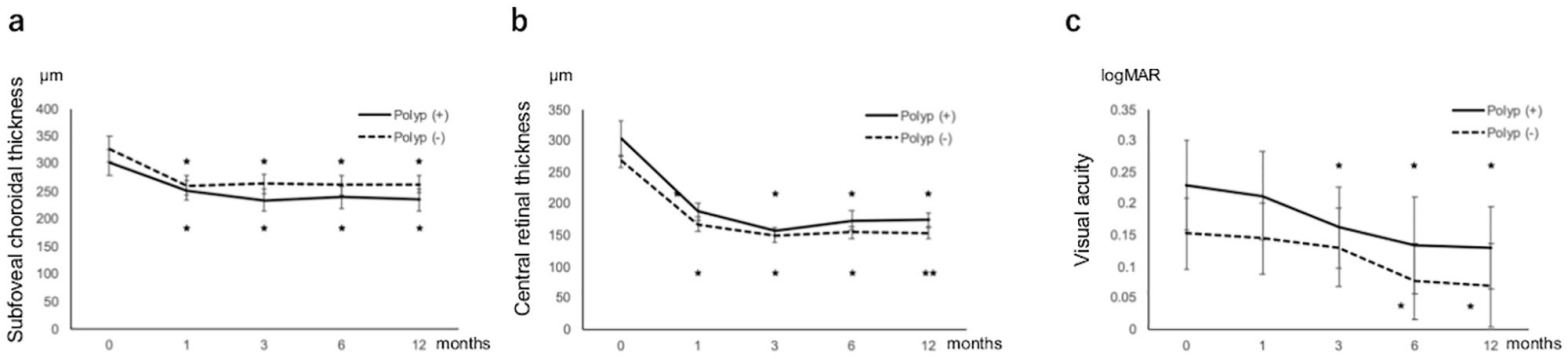
Changes in subfoveal choroidal thickness (a), central retinal thickness (b), and best-corrected visual acuity (c) during the 12-month follow-up period in eyes with pachychoroid neovasculopathy with or without polypoidal lesions. Each eye was treated with photodynamic therapy combined with intravitreal injections of an anti-vascular endothelial growth factor agent. No statistically significant difference in subfoveal choroidal thickness, central retinal thickness, and best-corrected visual acuity was observed between the two groups at any time points. *P < 0.05, **P < 0.01, compared with baseline values. Data are expressed as mean ± standard error.

Of the 42 eyes under study, 22 eyes (52.4%) had polypoidal lesions at baseline. Table 1 shows the clinical characteristics of eyes with or without polypoidal lesions. No significant differences in any clinical characteristics were observed between eyes with or without polypoidal lesions. Fig 2 shows SCT, CRT, and BCVA changes in both groups. SCT and CRT were significantly reduced at 12 months in both the groups when compared with that at baseline (P < 0.05 in SCT and CRT). In accordance with the SCT and CRT reduction, BCVA significantly improved at 12 months in both the groups (P < 0.01). Between the two groups, no significant differences in SCT, CRT, or BCVA were observed at any time points.

Of the 22 eyes with polypoidal lesions, 9 (40.9%) required at least one additional treatment within 12 months following initial therapy. Of the 20 eyes without polypoidal lesions, 1 (5.0%) needed two additional intravitreal injections of an anti-VEGF agent for 12 months and no eye received additional PDT. The number of additional treatments was significantly lower in the eyes with PNV without polypoidal lesions when compared to those with polypoidal lesions (P < 0.05) (Table 2). There were no eyes that developed new polypoidal lesions during the follow-up period.

## Discussion

In this present study, we demonstrated the efficacy of PDT combined with anti-VEGF therapy in eyes with treatment-naïve PNV. Overall, the combined therapy resulted in an improvement in the anatomical and visual function with only 1.9 injections, including initial treatment. A previous report [11] demonstrated that a subgroup of eyes with CVH experienced nonresponsiveness to intravitreal aflibercept. There are several reports describing the clinical and genetic differences between patients with AMD and PNV [12, 13]. Hata et al. [14] documented a lower intraocular VEGF-A concentration in eyes with PNV than in eyes with t-AMD. Factors other than VEGF may be also involved in the pathogenic mechanism of PNV. In contrast, PDT is reportedly effective for patients with CSC in terms of subretinal fluid resolution and reduced choroidal thickness [15]. PDT has been reportedly useful for eyes with type 1 CNV with thickened choroid refractory to intravitreal anti-VEGF treatment for complete fluid resolution [16]. As PNV is defined as a type 1 CNV with pachychoroid, treating CNV and decreasing the choroidal thickness are essential for maintaining the initial treatment outcome. Furthermore, since adverse side effects after PDT, such as subretinal hemorrhage, can be reduced by concomitant intravitreal injections of anti-VEGF [17], initial combination therapy should be considered for PNV cases.

Jung et al. [18] examined the treatment outcome of intravitreal injections of aflibercept and ranibizumab monotherapy in eyes with PNV, not including PCV. The mean number of injections in their study was 3.5 in eyes treated with aflibercept and 4.6 in eyes treated with ranibizumab during a 1-year follow-up. Furthermore, PDT was applied in 6 of the 54 eyes (11.1%) due to poor response. Chan et al. [19] explored the efficacy of PDT monotherapy (standard protocol) for CNV cases with CSC. They reported that three of seven eyes (43%) required additional PDT during a 1-year follow-up. Recently, reduced-fluence PDT has been applied as a treatment for CSC to reduce the complications associated with PDT. Smretschnig et al. [20] analyzed the treatment outcome of intravitreal anti-VEGF combined with half-fluence PDT for CNV in eyes with chronic CSC. In their study, 9 of 17 eyes (53%) received additional treatment during a 1-year follow-up. Another study by Matsumoto et al. [21], 4 of 21 eyes (19%) requiring additional therapy after intravitreal aflibercept combined with half-dose PDT in eyes PNV without polypoidal lesions. In this present study, the total number of injections was 1.1 in eyes with PNV without polypoidal lesions, and only one eye required additional treatment. All of these results indicate that intravitreal anti-VEGF injections combined with full-dose PDT could maintain the therapeutic outcome for a more extended time period and lessen the number of injections, thus reducing the treatment and economic burden for patients.

In the present study, there were significant visual and anatomical improvements in PNV cases with polypoidal lesion. The EVEREST II study [7] demonstrated the efficacy of intravitreal ranibizumab combined with PDT for eyes with PCV in terms of the number of injections and visual gain. The combination therapy group received a median of 4.0 ranibizumab injections compared with the monotherapy group, which received 7.0, for 12 months. Yanagi et al. [22] showed that eyes with PCV with CVH required fewer injections with better visual outcomes after initial combination therapy. The number of injections after initial combination therapy was 2.58 in the CVH (+) group, whereas that in the CVH (−) group was 4.68 during 1-year follow-up. In this present study, the mean number of injections for eyes with PNV with polypoidal lesions was 2.5, which was comparable with that reported by a previous report by Yanagi et al. [22]. While the protocol of the EVEREST II study comprised three consecutive intravitreal ranibizumab injections, our study consisted of a single intravitreal anti-VEGF injection before advancing to a PRN regimen. Therefore, the total number of injections in our study was different from that of the EVEREST II study. Considering the results of the study by Yanagi et al. [22], 3 months of intravitreal anti-VEGF injections may not be required for PNV eyes when combined with PDT. These results suggest that the combination therapy may be useful in reducing the number of injections and providing visual gain in eyes with PNV with polypoidal lesions.

No significant differences were observed in the changes in BCVA, CRT, and SCT values during the 1-year follow-up period between eyes with PNV, with and without polypoidal lesions. However, eyes with PNV with polypoidal lesions required significantly more injections than eyes with PNV without polypoidal lesions. The recurrence rate was higher in PNV cases with polypoidal lesions than that in cases without polypoidal lesions. PDT treatment has been reported to be effective for chronic CSC in complete resolution of the subretinal fluid [23]. Moreover, PNV cases without polypoidal lesions have clinical characteristics similar to those of CSC. Therefore, combination therapy may have a longer therapeutic effect in PNV cases without polypoidal lesions than in PNV cases with polypoidal lesions.

Our study has several limitations, including its retrospective nature and a relatively small number of subjects. BCVA was unlikely to be obtained in a systematic fashion because of the retrospective nature of this study. To date, the criteria of PNV are not clearly defined. Several researchers included PNV with polypoidal lesions in their inclusion criteria [14, 24]. Both PNV with and without polypoidal lesions are believed to occur due to pachychoroid-driven pathophysiological mechanisms. Therefore, we included both eyes with PNV, with and without polypoidal lesions in our study. We also did not consider choroidal thickness because CNV develops at the site of pachyvessels with inner choroidal attenuation, irrespective of choroidal thickness. As data for this study was collected from multiple clinical centers, different OCT systems were used for measuring CRT and SCT. Two different anti-VEGF agents were used for intravitreal injections. The choice of anti-VEGF agents and retreatment with or without PDT was made according to physicians’ discretion. Future studies with a larger sample size are required in order to evaluate the appropriate treatment protocol for eyes with PNV.

In conclusion, PDT combined with anti-VEGF therapy was effective for visual and anatomical improvements requiring a small number of additional treatments for eyes with PNV over a 1-year follow-up. The combination therapy could be the first choice for treating eyes with PNV in terms of reducing the treatment and economic burden for patients. There were no significant differences in visual and anatomical improvements between eyes with PNV with or without polypoidal lesions, whereas the recurrence rate was higher in the PNV cases with polypoidal lesions than in those without polypoidal lesions. A longer therapeutic effect may be obtained in eyes with PNV without polypoidal lesions.

## Supporting information

S1 Data (XLSX)
